# Age in combination with gender is a valuable parameter in differential diagnosis of solid pseudopapillary tumors and pancreatic neuroendocrine neoplasm

**DOI:** 10.1186/s12902-022-01164-7

**Published:** 2022-10-21

**Authors:** Leshuang Wu, Tianle Zou, Dan Shi, Huan Cheng, Muhammad Shahbaz, Muhammad Umar, Tianfeng Li, Xianbin Zhang, Peng Gong, Yushan Wei

**Affiliations:** 1 Department of Scientific Research, First Affiliated Hospital of Dalian Medical University, Dalian, China; 2 Department of Preventive Medicine, School of Public Health, Dalian Medical University, Dalian, China; 3 Department of General Surgery and Institute of Precision Diagnosis and Treatment of Gastrointestinal Tumors, Shenzhen University General Hospital and Shenzhen University Clinical Medical Academy, Shenzhen, China; 4 Carson International Cancer Center and Guangdong Provincial Key Laboratory of Regional Immunity and Diseases, Shenzhen University Health Science Center, Shenzhen, China; 5 Department of Healthcare ManagementSchool of Health management, China Medical University, Shenyang, China

**Keywords:** Solid pseudopapillary tumor, Pancreatic neuroendocrine neoplasm, Nomogram

## Abstract

**Background:**

The clinicopathological characteristics of solid pseudopapillary tumor (SPT) and pancreatic neuroendocrine neoplasm (pNEN) are different. We, therefore, systematically investigated the performance of the clinicopathological characteristics in distinguishing SPT from pNEN.

**Methods:**

We collected the cases from the Surveillance, Epidemiology, and End Results Program. The International Classification of Diseases for Oncology, third edition (ICD-O-3) for tumors was used to identify patients with pNEN or patients with SPT. To determine the performance of age in combination with gender in distinguishing SPT from pNEN, a nomogram was developed and the performance of this nomogram was evaluated by the receiver operating characteristic curve and the area under the curve (AUC).

**Results:**

In the training cohort, 563 patients with pNENs and 30 patients with SPTs were recruited. The logistic regression and receiver operating characteristic curves suggest that age, gender, T-stage, N-stage, and M-stage could discriminate SPT and pNEN. The AUC of age, gender, T-stage, N-stage, and M-stage was 0.82, 0.75, 0.65, 0.69, and 0.70, respectively. Based on the nomogram, we observed that the AUC of age and gender is significantly high than that of the T-stage, N-stage, and M-stage.

**Conclusions:**

The present study proposes a non-invasive nomogram that could aid in the differential diagnosis of pNEN and SPT. This might help the clinicians to distinguish SPT from pNEN and choose the appropriate treatments for the patients.

## Introduction

Pancreatic neuroendocrine neoplasm (pNEN) is rare and it is around 10 per million in 2015, which accounts for only 5.2% of all pancreatic neoplasms [1, 2]. In the last two decades, in order to describe these tumors, various nomenclatures were used, for example insulinoma, somatostatinoma, and gastrinoma, and until 2010 the World Health Organization (WHO) defined these tumors as neuroendocrine neoplasm [3]. Based on the histopathlogy and Ki-67 index, the pNENs were divided into well-differentiated neuroendocrine tumors (NETs) and poorly differentiated neuroendocrine carcinomas (NECs) [4]. In addition, according to the clinical manifestations, the pNENs are classified into functioning or nonfunctioning neoplasms. Functioning pNENs are characterized with specific symptoms, such as Zollinger-Ellison syndrome, wihich are caused by hormones. Nonfunctioning pNENs may also secrate hormones, however, it cannot lead to symptoms [5].

Solid pseudopapillary tumor (SPT) is a type of neoplasm whose incidence is lower than pNEN and it accounts for only 0.1% of all pancreatic tumors [6]. Same to pNEN, the nomenclature vasried in the last decades and until 1996 the WHO defined these tumors as SPTs and pathologically classified them as rare cystic pancreatic neoplasm [7]. In contrast to pNEN, which originates from pancreatic duct pluripotent stem cells, SPT may be derived from pancreatic embryonic stem cells. The 5-year survival rate of pNEN and SPT is 45.2% and 87.2%, respectively [2]. This suggests that the prognosis of SPT is better than pNEN. In addition, the management of these two types of pancreatic tumors is different. For example, due to the excellent prognosis and low malignant biological behavior of SPT, patients with advanced or metastatic tumors could achieve long-term survival and, therefore, aggressive surgical resection is a favorable intervention for these patients [8]. However, for pNEN, aggressive surgical resection is recommended for selected individuals with well-differentiated tumors [9].

Based on the aforementioned difference between pNEN and SPT, the accurate diagnosis of these two tumors is essential. Some studies reported that pNEN gives low signal intensity on T1-weighted imaging and SPT gives high signal intensity [10, 11]. Thus, magnetic resonance imaging (MRI) could be used to discriminate pNEN from SPT. It is noteworthy that pNEN might give a hypo-enhancement signal [12]. In addition, both pNEN and SPT could present calcification and cystic degeneration [8]. Therefore, differential diagnosis of pNEN and SPT is challenging when the atypical characteristics are found in these two types of tumors. Some studies prove that the clinical characteristics of pNEN and SPT are different [2, 13]. However, as far as we know, no study evaluated the performance of the clinicopathological characteristics in distinguishing SPT from pNEN. We, therefore, sought to evaluate the diagnostic performance of clinicopathological characteristics and develop a non-invasive clinical nomogram for discriminating pNEN from SPT.

## Patients and methods

### Inclusion and exclusion criteria

**Fig. 1:**
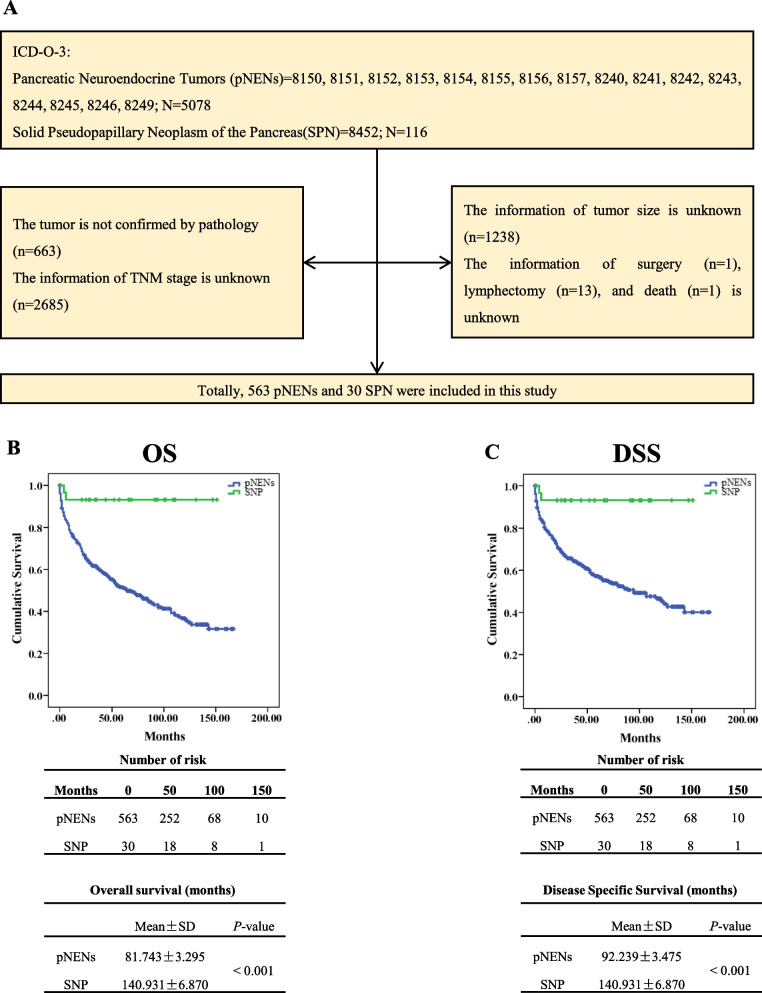
The flowchart of patient selection and the prognosis of patients. We collected the cases from SEER databases. Finally, 563 patients with pNEN and 30 patients with SPT were included in the present study (**A**). Compared to pNEN, SPT significantly increased the probability of survival (**B**) and significantly decrease the hazard of death (**C**). SEER: Surveillance, Epidemiology, and End Results; pNEN: pancreatic neuroendocrine neoplasm; SPT: pseudopapillary tumor

We systematically searched the Surveillance, Epidemiology, and End Results database. As indicates in Fig. 1A, the International Classification of Diseases for Oncology, third edition (ICD-O-3) for tumors was used to identify patients with pNENs (8150, 8151, 8152, 8153, 8154, 8155, 8156, 8157, 8240, 8241, 8242, 8243, 8244, 8245, 8246, 8249; *N*=5078) or patients with SPTs (8452; *N*=116). Subsequently, we excluded cases in which the tumors were not diagnosed by pathology (*n*=663), the information on TNM stage (American Joint Committee on Cancer staging classification, 7^th^ edition, *n*=2685), tumor size (*n*=1238), surgery (*n*=1), lymphadenectomy (*n*=13), and survival status were unclear (*n*=1) (Fig. 1A). Totally, five hundred and ninety-three patients, sixty seven pNEN patients and seven SPT patients, were enrolled in the training cohort.

### Statistical analysis

**Fig. 2:**
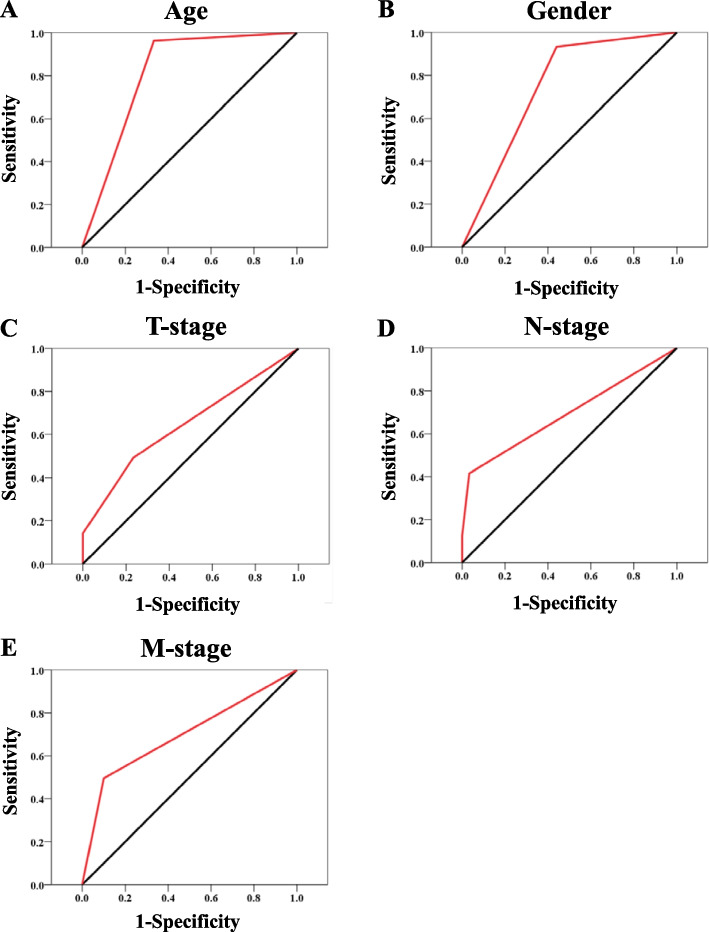
Evaluating the diagnostic performance of clinical characters. We performed the ROC curve and observed that age (**A**), gender (**B**), T-stage (**C**), N-stage (**D**), and M-stage (**E**) could distinguish pNENs from SPTs. pNEN: pancreatic neuroendocrine neoplasm; SPT: pseudopapillary tumor

**Fig. 3:**
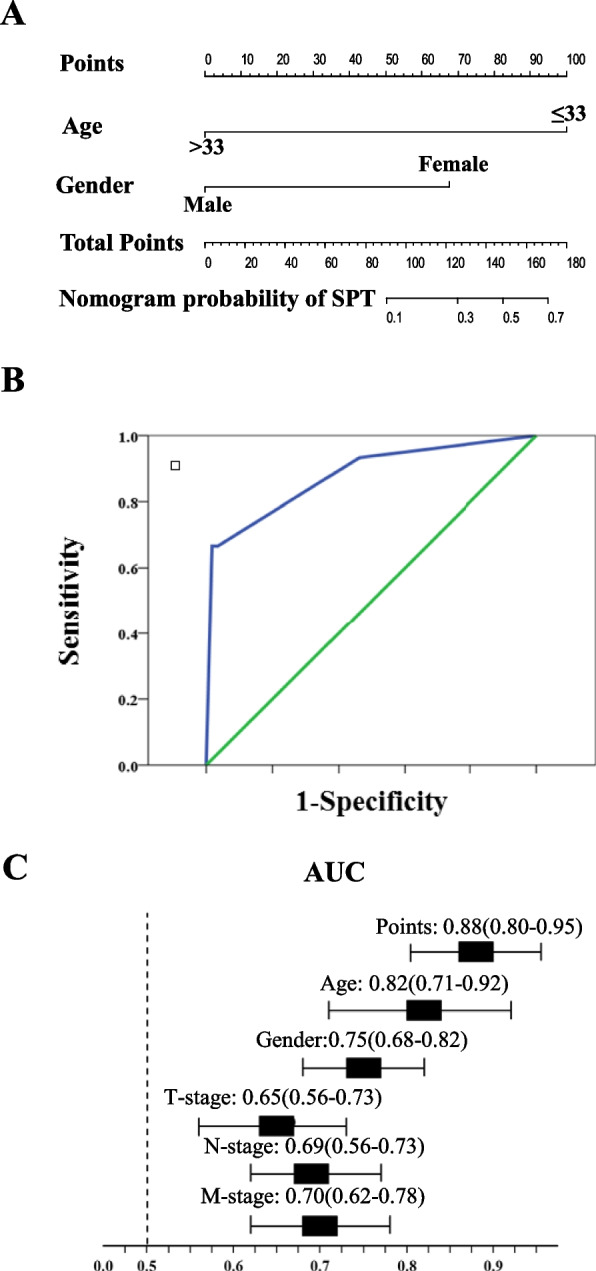
Development of a non-invasive nomogram and evaluating the diagnostic performance of this nomogram. Based on the age and gender, we developed a non-invasive nomogram (**A**) and the AUC of this nomogram was significantly high than that of the T-stage, N-stage, and M-stage (**B** and **C**). AUC: area under the curve

The survival time of patients was presented by the Kaplan-Meier curve, and the statistically significant was determined by the log-rank test (Fig. 1B). To describe the distribution of the data, the mean value and standard deviation were used for continuous variables. If the data followed a standard normal distribution and the variance of the two groups was equal, the statistical significance was evaluated by the student t-test. For categorical variables, the data were presented as the number and the percentage of patients. The statistical significance of the differences was determined by Fisher's exact test or Pearson's chi-squared test (Table 1). To find out the variables which could be used to discriminate pNEN from SPT, the univariate logistic regression was performed and the variables with *P*-value < 0.05 were used to perform the multiple logistic regressions (Table 2). Subsequently, the sensitivity and specificity of age, gender, T-stage, N-stage, and M-stage were evaluated by receiver operating characteristic (ROC) curves (Fig. 2). All the statistical analysis and the graphs were performed by SPSS 19 (IBM SPSS Statistics), and the nomogram was created by R software using the ‘rms’ package (Fig. 3A).

**Table 1:** The clinicopathological characteristics of patients

	**pNEN (*N*=563)**	**SPT (*N*=30)**	***P*-value**
Age (Mean±SD, years)	57.40±16.61	33.30±15.15	< 0.001*
Gender			< 0.001^#^
Male	315 (56.0%)	2 (6.7%)	
Female	248 (44.0%)	28 (93.3%)	
Race			0.403^$^
White	419 (74.4%)	19 (63.3%)	
Black	51 (9.1%)	4 (13.3%)	
Other	93 (16.5%)	7 (23.3%)	
Location			0.304^$^
Head	160 (28.4%)	8 (26.7%)	
Body/Tail	267 (47.4%)	18 (60.0%)	
Other	136 (24.2%)	4 (13.3%)	
Tumor Size (mm)	41.07±30.77	35.33±28.61	0.318*
T-Stage			< 0.001^$^
T1-Stage	114 (21.3%)	2(6.7%)	
T2-Stage	172 (32.1%)	21(70.0%)	
T3-Stage	151(28.2%)	5(16.7%)	
T4-Stage	46(8.6%)	2(6.7)	
Tx-Stage	80 (14.2%)	0 (0.0%)	
N-Stage			< 0.001^$^
N0-Stage	329 (58.4%)	29 (96.7%)	
N1-Stage	163 (29.0%)	1 (3.3%)	
Nx-Stage	71 (12.6%)	0 (0.0%)	
M-Stage			< 0.001^$^
M0-Stage	284 (50.4%)	27 (90.0%)	
M1-Stage	279 (49.6%)	3 (10.0%)	
AJCC-Stage			< 0.001^$^
I-Stage	170 (30.2%)	23 (76.7%)	
II-Stage	101 (17.9%)	4 (13.3%)	
III-Stage	13 (2.3%)	0 (0.0%)	
IV-Stage	292 (49.6%)	3 (10.0%)	
Grade			0.019^$^
Well differentiated	210 (37.3%)	5 (16.7%)	
Moderately differentiated	75 (13.3%)	4 (13.3%)	
Poorly differentiated	35 (6.2%)	0 (0.0%)	
Undifferentiated	14 (2.5%)	0 (0.0%)	
Unknown	229 (40.7%)	21 (70.0%)	

*pNEN* Pancreatic neoroendocrine neoplasm, *SPTs* Solid pseudopapillary tumor

^*^Student-t test

^#^Fisher exact test

^$^Pearson chi-squared test; *AJCC* American Joint Committee on Cancer

**Table 2:** Univariate and multivariate logistic regression

**Variable**	**Univariate Logistic Regression**			**Multivariate Logistic Regression**		
	**OR**	**β**	***P*-value**	**OR**	**β**	***P*-value**
**Age**						
< 33 years	Reference			Reference		
**≥33 years**	**0.019**	**-3.944**	**< 0.001**	**0.012**	**-4.386**	**< 0.001**
**Gender**						
Male	Reference				Reference	
**Female**	**17.782**	**2.878**	**< 0.001**	**14.247**	**2.657**	**0.002**
Race						
White	Reference				----	----
Black	1.730	0.548	0.336		----	----
Other	1.660	0.507	0.267		----	----
Location						
Head	Reference				----	----
Body/ Tail	1.348	0.299	0.494		----	----
Tumor size						
< 36 mm	Reference				----	----
≥36 mm	-0.079	-0.079	0.834		----	----
**T-Stage**						
T1-Stage	Reference				Reference	-----
**T2-Stage**	**6.959**	**1.940**	**0.010**	**28.840**	**3.362**	**0.001**
T3-Stage	1.887	0.635	0.453	**18.830**	**2.935**	**0.010**
**T4-Stage**	2.478	0.908	0.371	**196.699**	**5.282**	**< 0.001**
**N-Stage**						
N0-Stage	Reference				Reference	
**N1**-Stage	**0.070**	**-2.665**	**0.009**	**0.055**	**-2.902**	**0.012**
**M-Stage**						
M0-Stage	Reference				Reference	-----
**M1**-Stage	**0.113**	**-2.179**	**< 0.001**	**0.150**	**-1.898**	**0.027**
**AJCC-Stage**						
I-Stage	Reference			----	----	----
**II**-Stage	**0.293**	**-1.229**	**0.027**	----	----	----
III-Stage	**<**0.001	-19.203	0.999	----	----	----
**IV**-Stage	**0.079**	**-2.532**	**< 0.001**	----	----	----
Grade						
Well differentiated	Reference			----	----	----
Moderately differentiated	2.240	0.806	0.238	----	----	----
Poorly differentiated	**<**0.001	-17.465	0.998	----	----	----
Undifferentiated	**<**0.001	-17.465	0.998	----	----	----

*OR* Odds ratio, *AJCC* American Joint Committee on Cancer

## Results

### Clinical characteristics of pNEN and SPT

Five hundred and ninety-three patients with pNENs and 30 patients with SPTs were enrolled in the training cohort (Fig. 1A). We observed that the overall survival of patients with SPTs was significantly longer than that of patients with pNENs (SPTs vs. pNENs: 140.931 ± 6.810 vs. 81.743 ± 3.295; *P* < 0.001; Fig. 1B). To determine the probability of death caused by pNENs or SPTs, the cumulative hazard of death was calculated and we found that pNENs significantly increased the hazard of death and reduced the survival of patients (pNENs vs. SPTs: 92.239 ± 3.475 vs. 140.931 ± 6.870; *P* < 0.001; Fig. 1C). In addition, we observed that the age of patients with SPTs was significantly younger than that of patients with pNENs (SPTs vs. pNENs: 33.30 ± 15.15 vs. 57.40 ± 16.61; *P* < 0.001) and 93.3% of the SPTs patients were female (Table 1). Moreover, compared to SPTs, pNENs have advanced T-stage, N-stage, and M-stage (Table 1).

### The performance of clinical characteristics for differential diagnosis of pNENs and SPTs

To determine the clinical characteristics which could be used to discriminate pNENs from SPTs, we performed the univariate and multivariate logistic regression. We observed that old age (age≥33 years), male, T-stage, advanced N1-stage, and advanced M1-stage were the independent risk of pNENs. To evaluate the performance of these characteristics for differential diagnosis of pNENs and SPTs, we performed the ROC curve and we observed that the area under the curve (AUC) of age, gender, T-stage, N-stage, and M-stage was 0.82 (95%CI: 0.71-0.92, *P*<0.05; Fig. 2A), 0.75 (95%CI: 0.68-0.82, *P*<0.05; Fig. 2B), 0.65 (95%CI: 0.56-0.73, *P*<0.05; Fig. 2C), 0.69 (95%CI: 0.62-0.77, *P*<0.05; Fig. 2D), and 0.70 (95%CI: 0.62-0.78, *P*<0.05; Fig. 2E), respectively. This suggests that age, gender, T-stage, N-stage, and M-stage might be valuable clinical characters that could be used to distinguish pNENs from SPTs.

### Development and validation of a non-invasive nomogram for the diagnosis of pNENs and SPTs

To integrate the aforementioned variables and establish a non-invasive tool, which could help the clinicians with differential diagnoses of pNENs and SPTs, we developed a nomogram based on age and gender (Fig. 3A). To investigate the performance of this non-invasive nomogram, we performed the ROC curve (Fig. 3B) and determined the AUC of this nomogram, we observed that the AUC of this nomogram (AUC: 0.88; 95%CI: 0.80-0.95; Fig. 3C) was significantly high than that of T-stage (AUC: 0.65; 95%CI: 0.56-0.73), N-stage (AUC: 0.69; 95%CI: 0.62-0.77), and M-stage (AUC: 0.70; 95%CI: 0.62-0.78).

## Discussion

In the present study, we observed that age, gender, T-stage, N-stage and M-stage were valuable clinical characters that could be used to distinguish pNENs from SPTs. However, in order to obtaine the information of T-stage, N-stage and M-stage, the tumor tissues should be isolated from the pancrease by invasonal technologies, such as ultrasound-guided fine-needle biopsy (EUS-FNB) or surgical resection. We, thererfore, excluded these variabes from the present study and developed a non-invasive nomogram by age and gender. As indicated in Fig. 3A, a female (68 points) and under the age of 33 years old (100 points) will have a score of 168 points, which predicts the probility of SPT is 70%. This nomogram might have some clinical implications. For example, it might help the clinicians to accurately distinct pNENs from SPTs and to determine an appropriate diagnostic or treatment strategy for patients.

Although pNENs and SPTs are rare tumors, some studies suggested that the incidence of these tumors significantly increased in the last decade. Thus, pNENs and SPTs received attention in the publications [8]. As same as pNENs, SPTs patients have non-specific clinical manifestations, for example, abdominal pain, abdominal discomfort, and weight loss [14]. Usually, the patients are hospitalized due to abdominal masses or accidentally found a tumor in the pancreas. As presented in Fig. 1B, the survival of SPTs patients is significantly superior to pNENs. These observations are supported by other studies [15, 16]. In addition, compared to pNENs, the SPTs have relatively low malignant biological behavior. Thus, aggressive surgical resection might give rise to survival benefits in SPTs patients, even when patients with distant metastasis. Indeed, Wang et al. reported that surgical resections of the primary and metastatic lesions, as completely as possible, could give improve the prognosis of SPTs patients. However, surgical resection is contraindicated in patients with metastatic pNENs [17]. Therefore, an accurate preoperative diagnosis of SPTs and pNENs will help the clinicians to make optimal decisions and chose the appropriate treatments for SPTs and pNENs patients, respectively.As mentioned above, SPTs and pNENs have the same clinical symptoms and signs. In addition, the previous study reported that SPTs exhibit neuroendocrine differentiation and in these tumor tissues the author also observed chromogranin A, CA19-9, and vimentin which are used to diagnose pNENs [18]. This suggests that these tumor markers could not distinguish SPTs from pNENs. Notably, Li et al. evaluated the clinical and immunohistochemical characteristics of 37 SPTs, and they observed intracytoplasmic dot-like immunoreactivity of CD99 in these tumors [19]. This is in contrast to pNENs tumors in which the CD99 was observed in the membrane [19]. In addition, the authors found a loss of E-cadherin and aberrant nuclear expression of β-catenin in SPTs. Thus, the expression of CD99 in combination with E-cadherin and β-catenin might be valuable combinational tumor markers for the diagnosis of SPTs and pNENs.

Because sometimes both SPTs and pNENs have cystic degeneration and calcification, it is difficult to distinguish between SPTs and pNENs by computed tomography (CT). It is reported that MRI is a valuable strategy for the diagnosis of pancreatic tumors. Compared to CT, MRI could appropriately exhibit the soft-tissue characteristics, theretofore MRI could be used to evaluate the functional and metabolic of tumors. Notably, both SPTs and cystic endocrine tumors have the same features, such as areas of cystic change, enhancing components, and well-defined contours [20]. Although positron emission tomography/computed tomography (PET/CT) is widely used in the diagnosis of malignant tumors and pancreatic disease, there are very few studies that evaluated the accuracy of PET/CT in the diagnosis of SPTs [21–23]. François et al. reported that ^18^F-FDOPA PET/CT was a promising approach for the diagnosis of pNENs and SPTs. The ^18^F-FDOPA PET-positive/SRS-negative lesions might be the SPTs. However, these should be verified in a large cohort [21]. EUS-FNB might be another promising diagnostic tool for distincitng SPTs from PNENs [24, 25]. Recently, some studies reported that the diagnostic accuracy of EUS-FNB in combination with the immunohistochemical staining of β-catenin or cadherin in solid pancreatic lesions is high than 90% [26–28]. Therefore, EUS-FNB should be recommmeded as the standard of care for differential diagnosis of pancreatic lesions.

Notably, there are some limitations to our study. This is a retrospective study and the confounding bias might distort the association between the variables and SPTs. In addition, SPTs and pNENs are rare tumors. To collect as many patients as possible, we used the data in the Surveillance, Epidemiology, and End Results database which encompasses approximately 28% of the USA population. However, after a strict selection process, only 30 SPTs patients were included in the present study. In addition, based on the pathology and Ki-67 index, the grade of pNENs is classified into four groups: pNET G1 (Ki-67<2%), pNET G2 (3%<Ki-67<20%), pNET G3 (Ki-67>20%) and pNEC [4]. While this WHO classification and the Ki-67 index are not recorded in the SEER database, and the tumore is graded according to morphological description, for example, well differentiated, moderately differentiated and poorly differentiated. Moreover, 40.7% cases lost the information of the tumor grade. These limitations of SEER database might also cause bias when interpreting the results.

## Conclusions

In summary, based on the age and gender of patients, we developed a non-invasive nomogram, which could discriminate SPTs and pNEN. This nomogram might help the clinicians appropriately diagnose SPTs and pNEN and chose the optimal strategies for these patients.

## Data Availability

The data and material are available from the corresponding author on reasonable request.
